# Delay in the Diagnosis of Adult-Onset Still’s Disease

**DOI:** 10.7759/cureus.1321

**Published:** 2017-06-07

**Authors:** Stella Pak, Cindy Pham

**Affiliations:** 1 Internal Medicine, University of Toledo Medical Center

**Keywords:** adult onset still’s disease, fever of unknown origin, anakinra

## Abstract

Adult-onset Still’s disease (AOSD) is a systemic autoinflammatory disease characterized by symptoms including spiking fever, arthralgia, myalgia, maculopapular rash, and pharyngitis. The lack of diagnostic biomarker, non-specific clinical presentation, and the rarity of AOSD often result in a significant delay in diagnosis and treatment. While the average time of initial presentation to diagnosis is four months, we present a case of AOSD diagnosis three years after initial onset of classical symptoms. By reporting the case of delayed diagnosis for AOSD, we hope to raise awareness in our medical community about the diagnostic difficulty in AOSD. The present case describes an otherwise healthy male who presented with typical symptoms of AOSD, but the diagnosis of AOSD was missed during his first presentation. In the second flaring episode, the diagnosis of AOSD was established. He had an excellent therapeutic response to anakinra and prednisone during the acute flaring episode. He is currently in complete remission on methotrexate as maintenance therapy.

## Introduction

Adult-onset Still’s disease (AOSD) is a systemic autoinflammatory disease characterized by symptoms including quotidian or double-quotidian spiking fever, arthralgia, myalgia, maculopapular rash, pharyngitis, anorexia, nausea and weight loss [1]. The incidence of AOSD is estimated to be about 0.16 case per 100,000 people [2]. The lack of serologic biomarker, non-specific clinical presentation and the rarity of AOSD often result in a significant delay in diagnosis and treatment. A recent retrospective study reported the mean time from initial presentation to diagnosis as four months [3]. Reported complications of AOSD include the following: fulminant hepatitis, myocarditis, acute respiratory distress syndrome, serositis, macrophage activation syndrome, disseminated intravascular coagulopathy, septic shock, pulmonary artery hypertension, diffuse alveolar hemorrhage, and thrombotic thrombocytopenia purpura [4]. Early diagnosis and treatment could lead to a favorable prognosis and prevention of severe complications. Thus, raising awareness of the disease and its diagnostic challenges is the first step towards addressing this issue. Early diagnosis and intervention may help prevent the development of complication and improve the patient outcomes. Therefore, an urgent change is warranted to address the diagnostic dilemma in AOSD. By reporting the case of a delayed diagnosis for AOSD, we hope to raise awareness in our medical community about the diagnostic difficulty in AOSD. Informed consent statement was obtained for this study.

## Case presentation

A 31-year-old male with no past medical history of illness presented to the emergency department (ED) with persistent spikes of fever (102 °F) and chills for the past two weeks. The onset of symptoms occurred immediately following his return from a trip to Mexico. His fever and chills were associated with abdominal pain, anorexia, night sweats, dyspnea, headache, myalgia, and fatigue. Symptoms were preceded by a reddish-purple edematous and tender lesion on his right foot.

Extensive workup involving imaging and serology during hospital admission and outpatient follow-ups were non-revealing. Chest X-ray and computerized tomography (CT) of chest, abdomen, and pelvis did not show any abnormality. Complete blood count (CBC), comprehensive metabolic panel (CBP), fungal serology, blood culture, histoplasma antigen test and rapid influenza diagnostic test were within normal limit. The only abnormal findings from laboratory investigation were total bilirubin of 2.1 mg/dL, C-reactive protein (CRP) of 1.9 mg/L and erythrocyte sedimentation rate (ESR) of 12 mm/hr. The patient was discharged with ciprofloxacin for a possible gastrointestinal (GI) infection. In the outpatient follow-up, ultrasonographic examination of the liver, pancreas and spleen did not show any abnormal finding.

**Figure 1:**
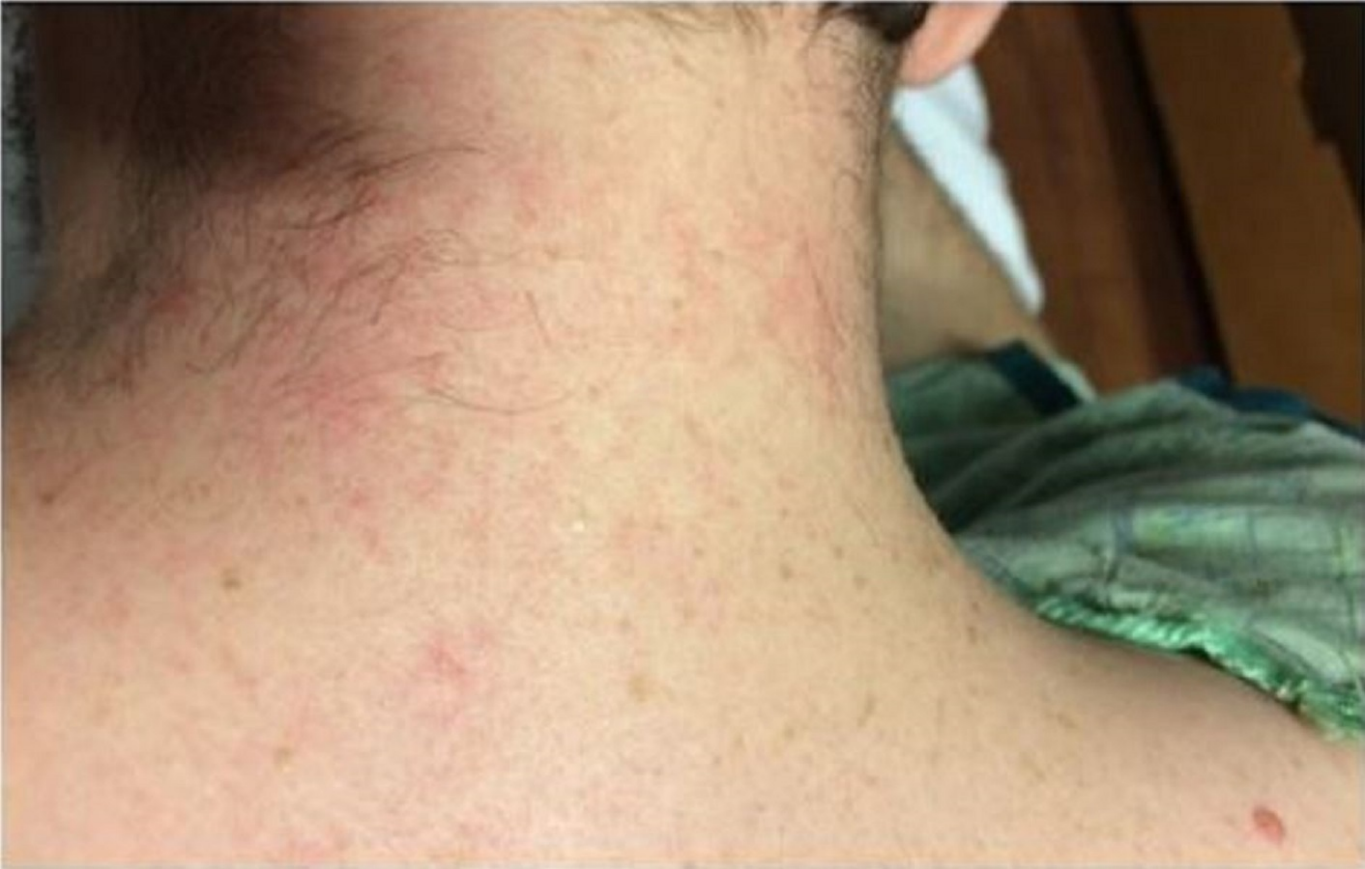
Maculopapular rash in the neck

**Figure 2:**
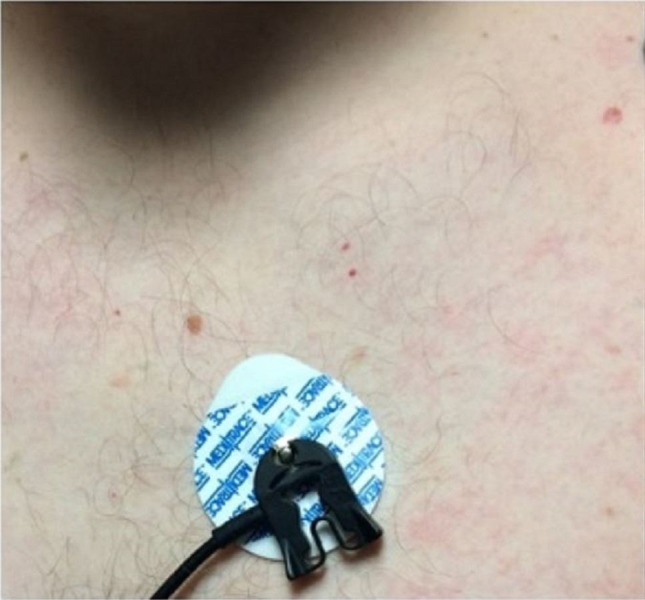
Maculopapular rash in the chest

The patient remained asymptomatic for three years before returning to the ED once again for persistent intermittent fever (103 °F) and chills for the past eight days. Without the use of acetaminophen, patient’s fever was determined to be quotidian in nature. Symptoms were associated with cyclic intractable headache, nausea, and vomiting, abdominal and back pain, and generalized arthralgia. Patient was admitted to the hospital and once again, repeat imaging (chest X-ray, CT scan of the chest and abdomen) and extensive infectious workup was non-revealing, including cerebrospinal fluid, urine, and stool studies; hepatitis panel, cytomegalovirus, Epstein-Barr virus, Toxoplasma, Bartonella test, human immunodeficiency virus, syphilis, lactate dehydrogenase, peripheral smear, and pan cultures. Significant laboratory findings were ferritin 490 ng/mL, ESR 23 mm/hr, CRP 23.7 mg/L, alanine transaminase (ALT) 55 U/L, and aspartate transaminase (AST) 40 U/L. Despite the use of ciprofloxacin, the patient remained cyclically febrile. At this point, rheumatology was consulted and noted a non-pruritic generalized erythematous maculopapular rash occurring only during febrile episodes (Figure 1 and 2). Autoimmune workup was unremarkable which included antinuclear antibodies (ANA), anti-neutrophil cytoplasmic antibodies (ANCA), complement 3 (C3), complement 4 (C4), and rheumatic factor (RF).

In the light of patient’s quotidian fever pattern, evanescent rash, arthralgia, elevated acute phase reactants, in addition to negative autoimmune and infectious workup, a preliminary diagnosis of Adult-onset Still’s disease was made. The patient responded well to two subcutaneous injections of anakinra 200 mg during his hospital stay with a decrease in febrile frequency. At the one-week follow-up visit, he was the transition from anakinra to prednisone 40 mg daily. Once patient became asymptomatic, he was slowly tapered off of steroids over the course of six months and then started on methotrexate 20 mg weekly. The patient continued to remain asymptomatic after nine months.

## Discussion

Adult-onset Still’s disease (AOSD) is a multi-systemic inflammatory disorder characterized by a triad of spiking the fever, salmon-colored rash, and arthritis. The name of the syndrome is derived from the fact that it resembles the systemic form of juvenile rheumatoid arthritis first described by Dr. Still, a renowned British pediatrician. It is found worldwide and predominantly affects young adults between the ages of 16 and 35 [1, 4].

Yamaguchi’s criteria are the most widely used and sensitive diagnostic tool for AOSD (4). Yamaguchi’s criteria require five or more criteria, including at least two major criteria for the classification of AOSD. Major criteria include the high fever of at least 39 °C for at least one week, arthralgia with a minimum duration of two weeks, skin rash, and neutrophilic leukocytosis (> 10,000/µL, ≥ 80% granulocytes). Minor criteria include ANA and RF negativity, sore throat, lymphadenopathy and/or splenomegaly and hepatic dysfunction [5]. Our patient presented with three of Yamaguchi major criteria - a high-grade fever of 102-103 °F for at least one week, arthralgia for two weeks, and skin rash. He also met two of Yamaguchi minor criteria - hepatic dysfunction and absence of ANA and RF. Thus, our patient fulfills Yamaguchi’s criteria for the diagnosis of AOSD.

Other laboratory tests reflecting heightened immunological activity besides leukocytosis and elevated acute phase reactants and particularly elevated serum ferritin levels. A high level of ESR and CRP was found in virtually all patients [6]. An elevated level of ferritin was reported in nearly 70% of patients with AOSD [4]. Our patient also had high levels of ferritin, ESR, and CRP. It has been shown that the combination of a glycosylated ferritin fraction less than 20% and ferritin levels above the upper limit of normal improved the sensitivity and specificity (to 70.5 % and 83.2%, respectively) as compared with using elevated ferritin levels alone [7]. In 2002, Fautre, et al. proposed a new criterion to account for the observation of elevated ferritin levels in the majority of patients with AOSD [8]. The sensitivity and specificity of Fautrel criteria were 80.6 % and 98.5%, respectively [7]. The Fautrel major criteria included the spiking fever of at least 39°C, arthralgia, transient erythema, pharyngitis, neutrophilic polymorphonuclear count ≥ 80%, and glycosylated ferritin fraction ≤ 20%. The Fautrel minor criteria include typical Still's rash and leukocytosis (10,000/mm^3^). Diagnosis of AOSD by Fautrel criteria requires four or more major criteria or three major and two minor criteria [8].

## Conclusions

In conclusion, our case underlines the diagnostic challenge of AOSD. The typical timeframe between the initial presentation and the diagnosis of AOSD is around four months. However, our patient was diagnosed three years after his initial onset of symptoms. As a result of the diagnostic delay, the patient suffered another episode of an acute flare and received inappropriate antibiotic therapy on two separate occasions. Multidisciplinary efforts among clinicians are warranted to address the diagnostic challenge for a timely diagnosis and treatment of AOSD to prevent complications and lead to a favorable prognosis with improved quality of life.
